# Supplementary material for: Carba-NAD binding activates SIR2 by reshaping conformational plasticity and rewiring long-range allosteric networks
Source: PLoS Comput Biol. 2026 Feb 20;22(2):e1013966. doi: 10.1371/journal.pcbi.1013966 (PMC12923025; doi:10.1371/journal.pcbi.1013966)
Supplement: S1 Text — Fig A: Molecular dynamics analyses of SIR2 in the apo and CNA-bound states. Fig B: Total Weight Distribution by Residue. Fig C: Betweenness Centrality Distribution by Residue. Fig D: Conformational stability of SIR2 in the unbound and NAD ⁺ -bound states. Fig E: Redistribution of conformational flexibility upon NAD⁺ binding. Fig F: Principal component and free energy landscape analyses of NAD ⁺ -bound SIR2. Fig G: NAD ⁺ -induced reorganization of the SIR2 residue interaction network. Fig H: NAD ⁺ -induced remodeling of shortest signal transduction pathways in SIR2. Fig I: Overall distribution of 15 potential binding pockets predicted by Fpocket. Fig J: Control NRI Model Analysis. Table A: Top ten small molecules ranked by molecular docking affinity and their statistical parameters. (DOCX) [file pcbi.1013966.s001.docx]

**Carba-NAD binding activates SIR2 by reshaping confo-rmational plasticity and rewiring long-range allosteric networks**

**
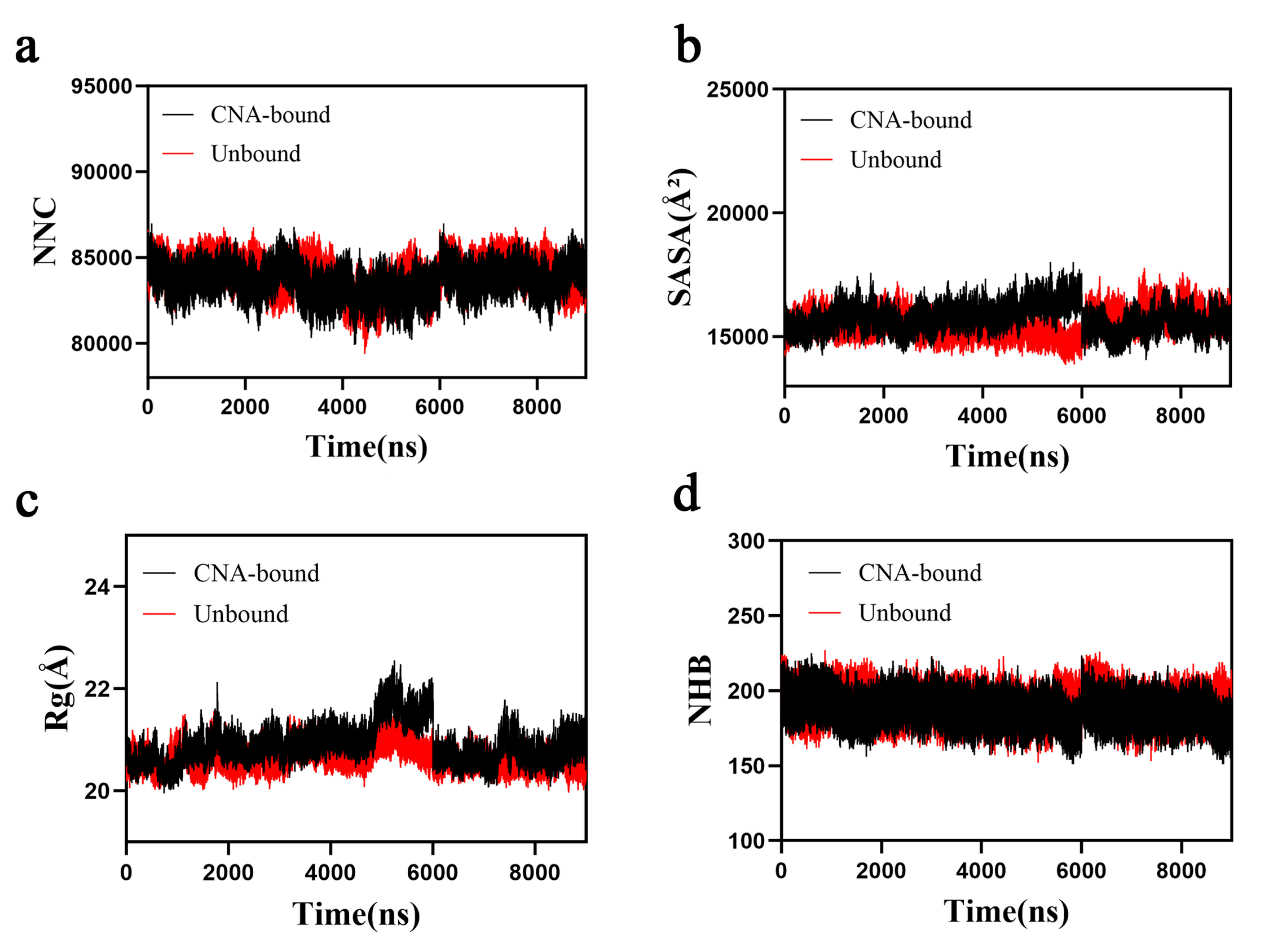
**

**Fig A in S1 Text:** **Molecular dynamics analyses of SIR2 in the apo and CNA-bound states.** (a) NNC, representing the number of native contacts; (b) SASA, solvent-accessible surface area; (c) Rg, radius of gyration; and (d) NHB, number of hydrogen bonds. Each panel compares the dynamics of SIR2 in the apo (red) and CNA-bound (black) states over the simulation trajectory, reflecting conformational changes induced by ligand binding.

**
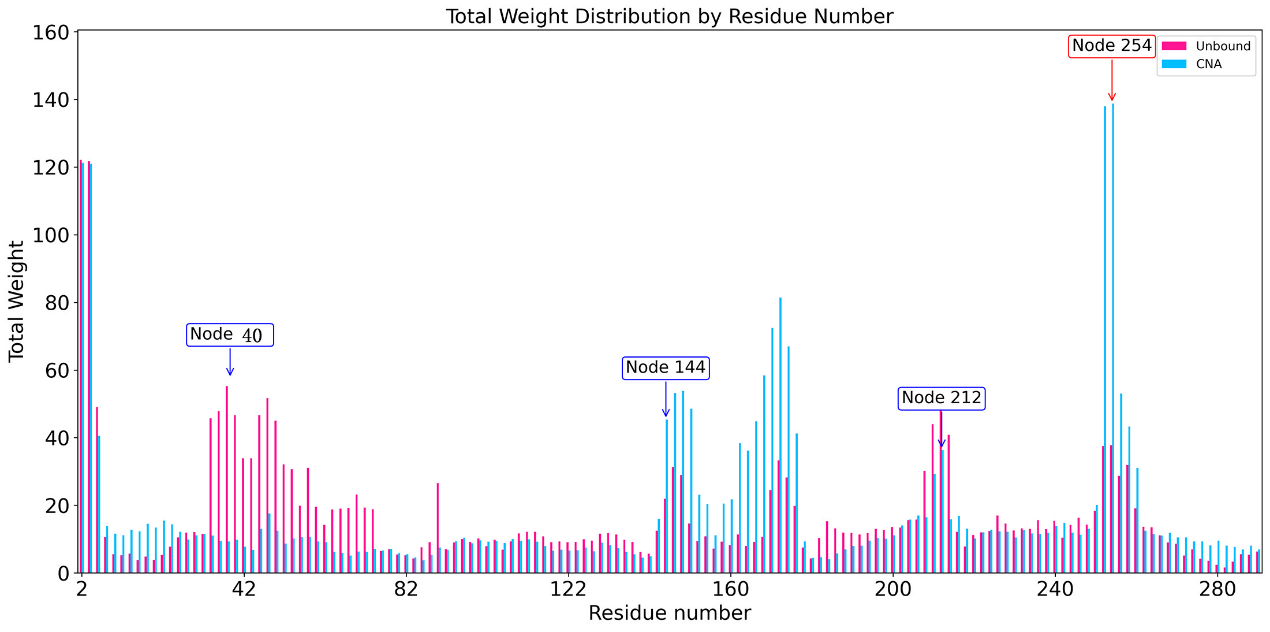
**

**Fig B in S1 Text: Total Weight Distribution by Residue.** The CNA-bound state (blue) exhibited a higher average weight (18.184 vs 17.299), the residues with the highest weights have been labeled.

**
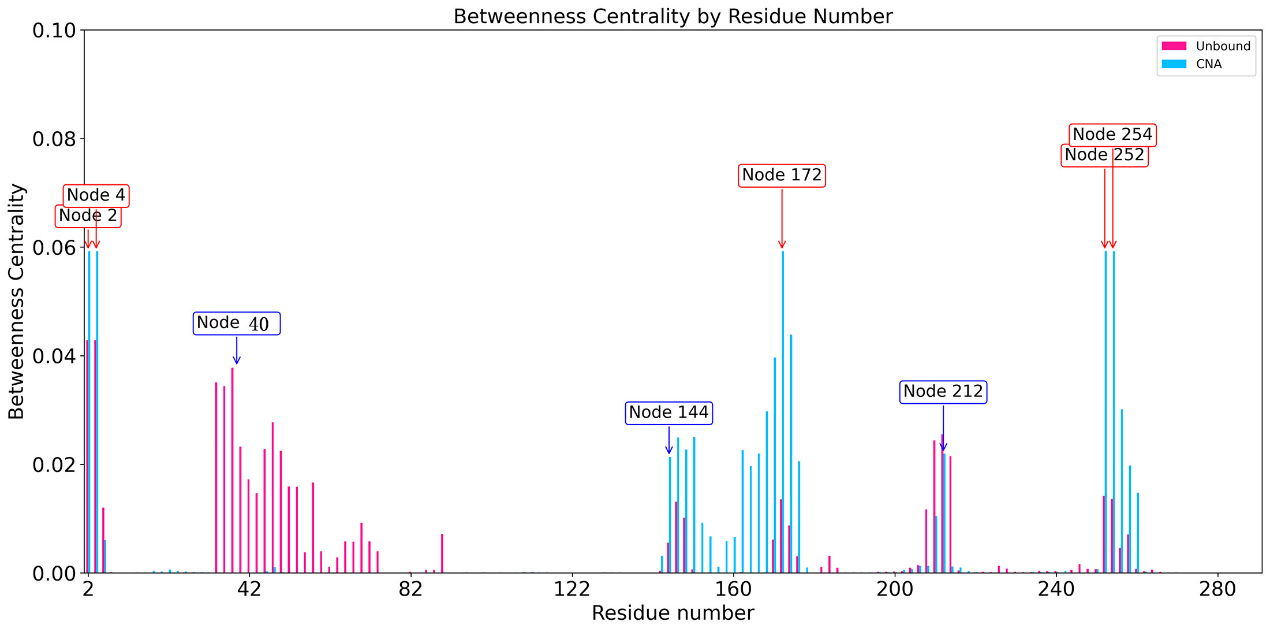
**

**Fig C in S1 Text: Betweenness Centrality Distribution by Residue.** Measures of betweenness centrality, such as average betweenness centrality (0.00505 vs 0.00440).

**Note 1 in S1 Text:: Control simulations with NAD⁺**

To verify that the mechanistic features uncovered with the non-hydrolyzable analog Carba-NAD (CNA) are not artifacts of analog substitution, we performed control simulations with the physiological cofactor NAD⁺. Three independent 3-μs trajectories were generated for the NAD⁺-bound system, yielding 9 μs of cumulative sampling. These simulations consistently reproduced the signatures of allosteric activation previously attributed to CNA binding. Specifically, RMSD analysis (Figure S3) confirmed that NAD⁺ binding allows stable sampling while enabling occasional conformational transitions. RMSF profiles (Figure S4) revealed rigidification of the catalytic β1–α2 loop accompanied by increased mobility in distal modules (S1–S5), establishing the same “rigid core–flexible periphery” organization as observed with CNA. Principal component and free energy landscape analyses (Figure S5) demonstrated an expansion of conformational space biased toward activation-aligned substates. At the network level, NRI-derived interaction maps (Figure S6) showed redistribution of centrality away from the β1–α2 loop and toward distal relay nodes such as S2 and S5. Shortest-path analysis (Figure S7) further corroborated these findings by revealing diversified communication routes connecting the catalytic core to remote loops.

Taken together, these results demonstrate that the local anchoring, distal releasing, network rewiring, and multi-path relay propagation observed in this study are intrinsic properties of the SIR2 scaffold. The convergence between NAD⁺- and CNA-bound ensembles strongly reinforces the robustness and generalizability of our proposed activation model.

**
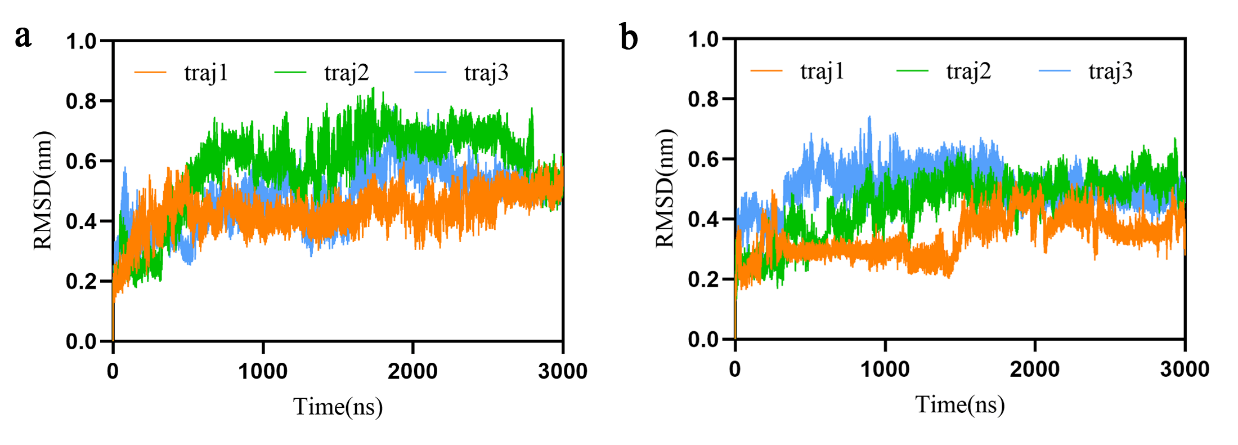
**

**Fig D in S1 Text: Conformational stability of SIR2 in the unbound and NAD⁺-bound states.** (a) Backbone RMSD profiles for the unbound system across three independent 3-μs trajectories. (b) Backbone RMSD profiles for the NAD⁺-bound system across three replicates. All simulations gradually reached equilibrium and maintained stable folds. The NAD⁺-bound ensemble displayed higher average RMSD values and occasional discrete jumps, suggestive of rare conformational transitions or substate switching. Together, these observations indicate that NAD⁺ binding expands the conformational landscape while preserving overall structural integrity. These trends are consistent with CNA-bound simulations, reinforcing the robustness of the mechanistic interpretation.


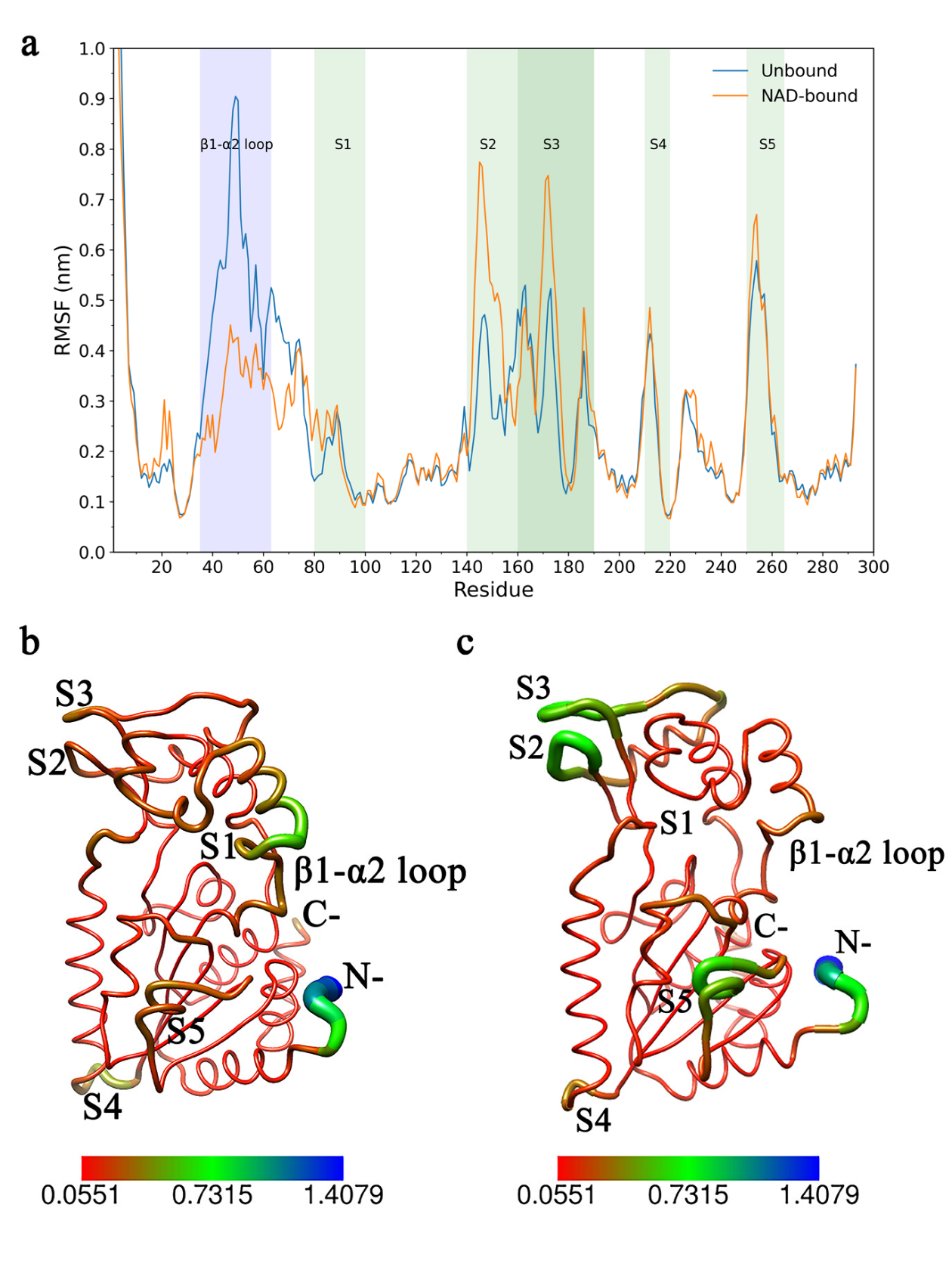


**Fig E in S1 Text: Redistribution of conformational flexibility upon NAD⁺ binding.** (a) Residue-wise RMSF profiles comparing the unbound (blue) and NAD⁺-bound (orange) systems. The β1–α2 loop (residues 35–63) is shaded in purple, showing marked rigidification upon NAD⁺ engagement. Five distal regions exhibiting enhanced fluctuations are defined as S1–S5 and highlighted in green bands. (b–c) RMSF values mapped onto the three-dimensional backbone structure of SIR2 for the unbound (b) and NAD⁺-bound (c) states. A color gradient from red (rigid) to blue (flexible) encodes fluctuation amplitude. In the NAD⁺-bound state, the β1–α2 loop condenses into a rigid conformation, whereas S1–S5 exhibit enhanced motional freedom, establishing a “rigid core–flexible periphery” organization. These NAD⁺-derived results parallel those observed with CNA, confirming that selective rigidification and distal flexibilization represent a generalizable allosteric signature.


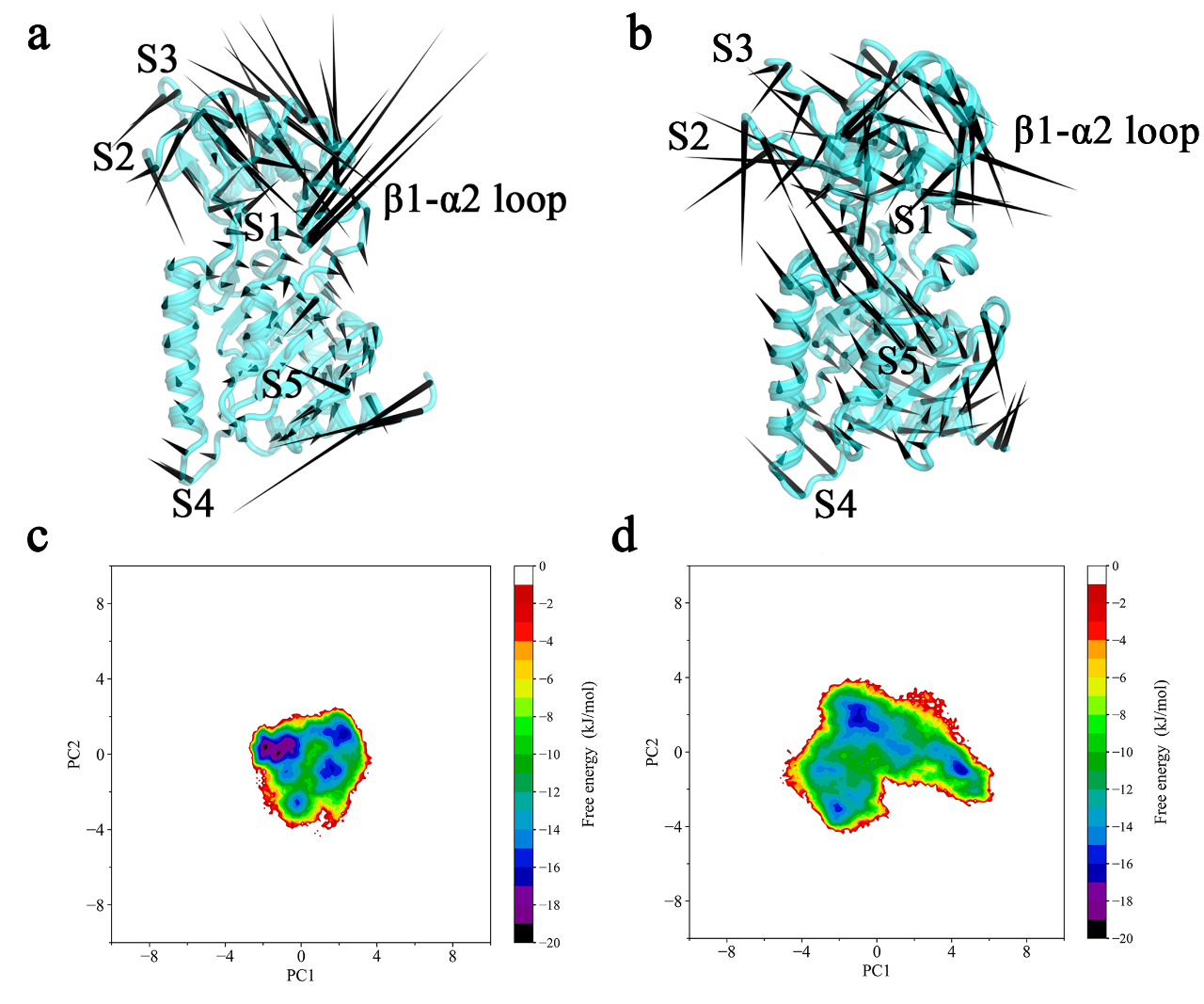


**Fig F in S1 Text: Principal component and free energy landscape analyses of NAD⁺-bound SIR2.** (a–b) Porcupine plots along the first principal component (PC1) for the unbound (a) and NAD⁺-bound (b) ensembles. Arrows depict dominant motions: the unbound state shows outward expansion of the β1–α2 loop (inactive geometry), whereas NAD⁺ binding drives loop closure coupled to outward displacement of S1–S5, manifesting a “loop-locking, periphery-releasing” pattern. (c–d) Free energy landscapes (FELs) projected onto PC1 and PC2 for the unbound (c) and NAD⁺-bound (d) states. The unbound FEL exhibits a narrow and deep basin, reflecting limited conformational sampling. In contrast, the NAD⁺-bound state displays broader and shallower basins elongated along PC1, indicative of directional bias toward activation-aligned conformations. These findings converge with CNA-bound results, underscoring that NAD⁺ binding not only broadens the thermodynamic ensemble but also actively steers it toward catalytically competent substates.


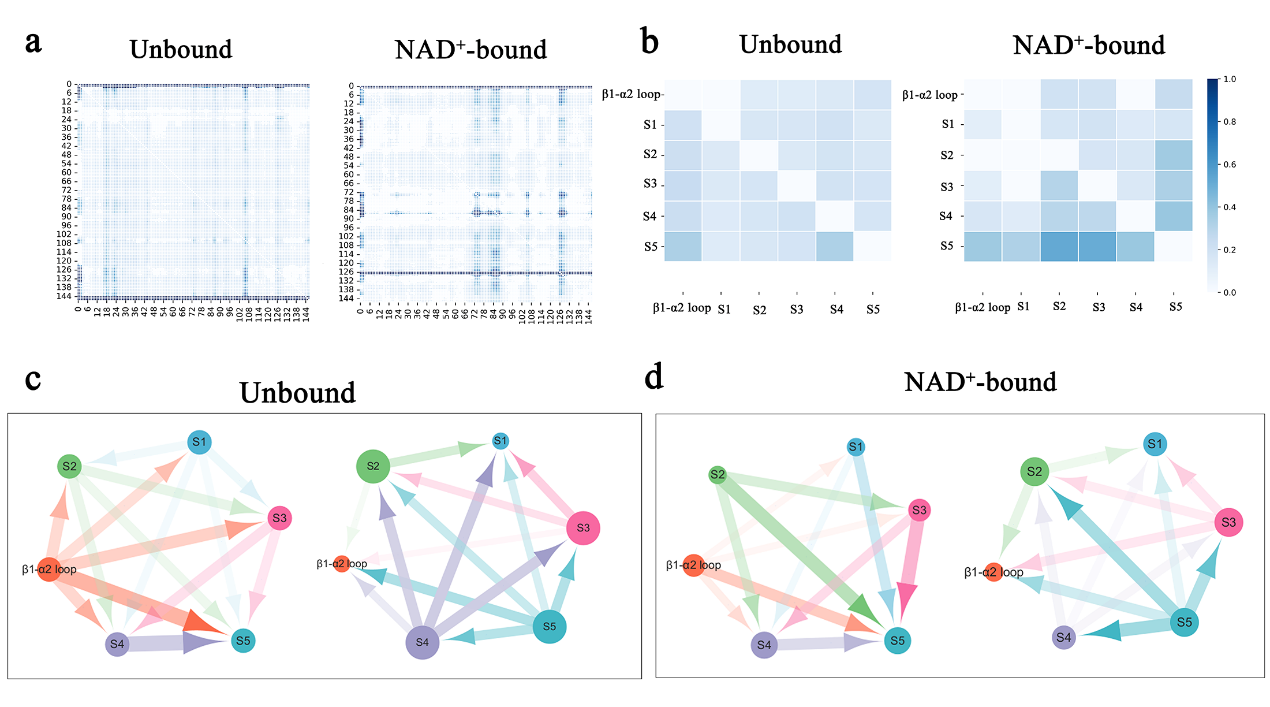


**Fig G in S1 Text: NAD⁺-induced reorganization of the SIR2 residue interaction network.** (a) Residue–residue interaction matrices inferred from NRI for the unbound (left) and NAD⁺-bound (right) ensembles. Color intensity indicates dynamic coupling probability, with darker blue reflecting stronger interactions. (b) Inter-domain communication strengths between the β1–α2 loop and distal modules (S1–S5). The unbound state is dominated by loop-centric influence, whereas NAD⁺ binding reduces its centrality and enhances distal relay, particularly via S2 and S5.

(c–d) Directed network topologies reconstructed for the unbound (c) and NAD⁺-bound (d) systems. Node size denotes total interaction strength; edge thickness encodes directional coupling intensity. The unbound ensemble follows a star-like topology with the β1–α2 loop as the broadcasting hub. In contrast, the NAD⁺-bound state exhibits a decentralized relay architecture in which S2 and S5 emerge as new hubs, redistributing control away from the catalytic loop. This architectural shift mirrors the “loop-locking, periphery-releasing” dynamic pattern, embedding catalytic precision at the core while enhancing distal adaptability. Importantly, these NAD⁺ results are consistent with CNA simulations, demonstrating that allosteric rewiring is an intrinsic property of the SIR2 scaffold rather than an artifact of using a cofactor analog.


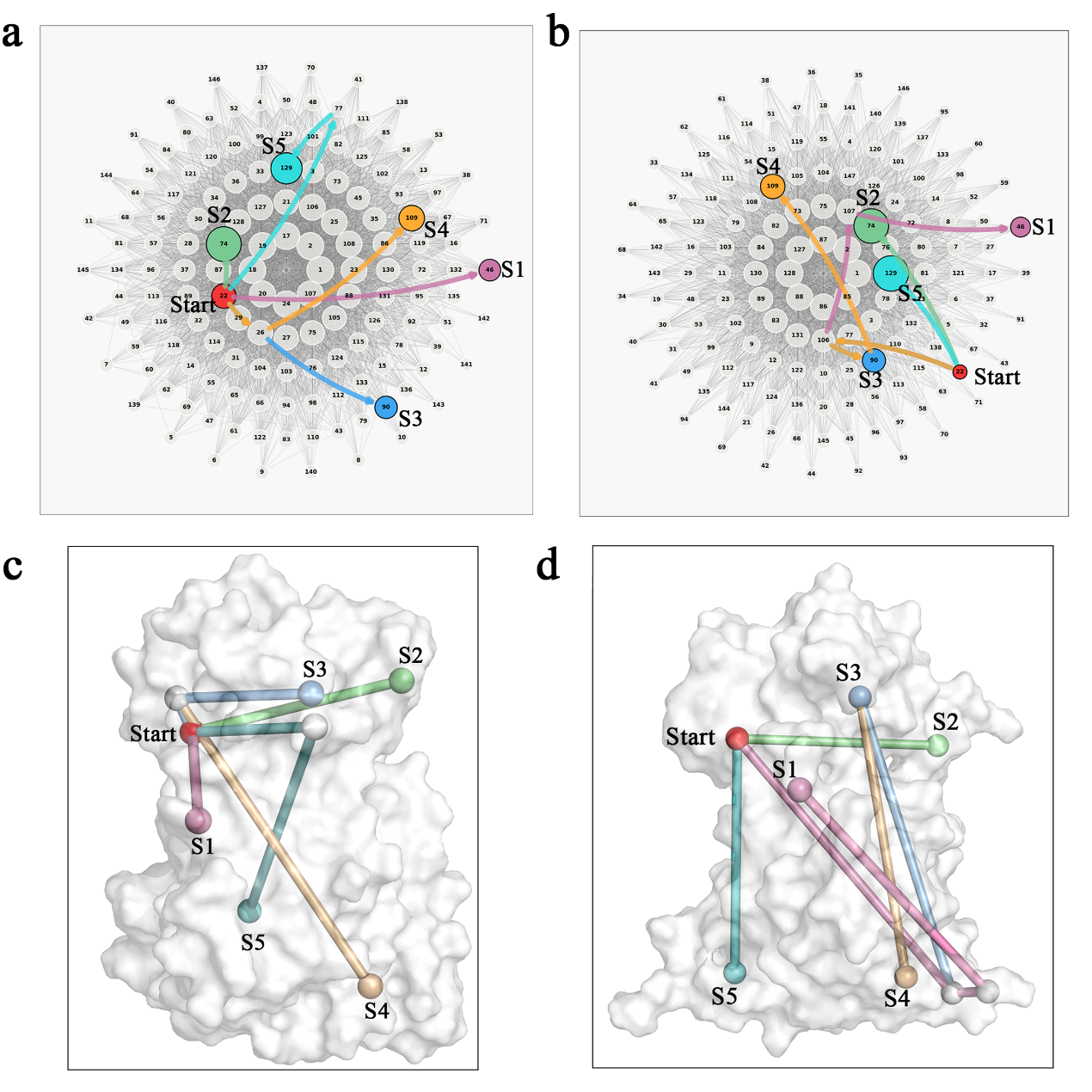


**Fig H in S1 Text: NAD⁺-induced remodeling of shortest signal transduction pathways in SIR2.** (a–b) Circular network layouts illustrating the shortest communication paths between the β1–α2 loop (red node, “Start”) and distal flexible regions S1–S5 in the unbound (a) and NAD⁺-bound (b) ensembles. Each distal region is color-coded (purple: S1, green: S2, blue: S3, orange: S4, cyan: S5). Nodes are arranged concentrically according to their centrality rank, with proximity to the center indicating greater regulatory influence; node size further encodes centrality. Edges depict residue–residue couplings along the minimal-resistance signal propagation routes, highlighting how NAD⁺ binding redistributes paths toward distal relay hubs (notably S2 and S5). (c–d) Three-dimensional mapping of the same shortest paths onto the molecular surface of SIR2 in the unbound (c) and NAD⁺-bound (d) states. The red sphere marks the geometric center of the β1–α2 loop, while colored spheres denote the centers of S1–S5. Paths are visualized as smoothed connections linking representative Cα atoms along each minimal route, maintaining the color scheme from (a–b). The NAD⁺-bound state exhibits expanded and diversified routes, forming multi-hop bridges that couple local rigidification at the catalytic loop with distal flexibility hotspots. Together, these analyses demonstrate that NAD⁺ binding reorganizes signal transduction from a loop-centric broadcast architecture to a distributed relay system mediated by distal modules. This decentralized routing provides a structural basis for robust long-range communication. Importantly, the NAD⁺ results mirror those obtained for CNA, confirming that the emergence of multi-path relay architecture reflects an intrinsic feature of the SIR2 scaffold rather than an artifact of using a cofactor analog.

**
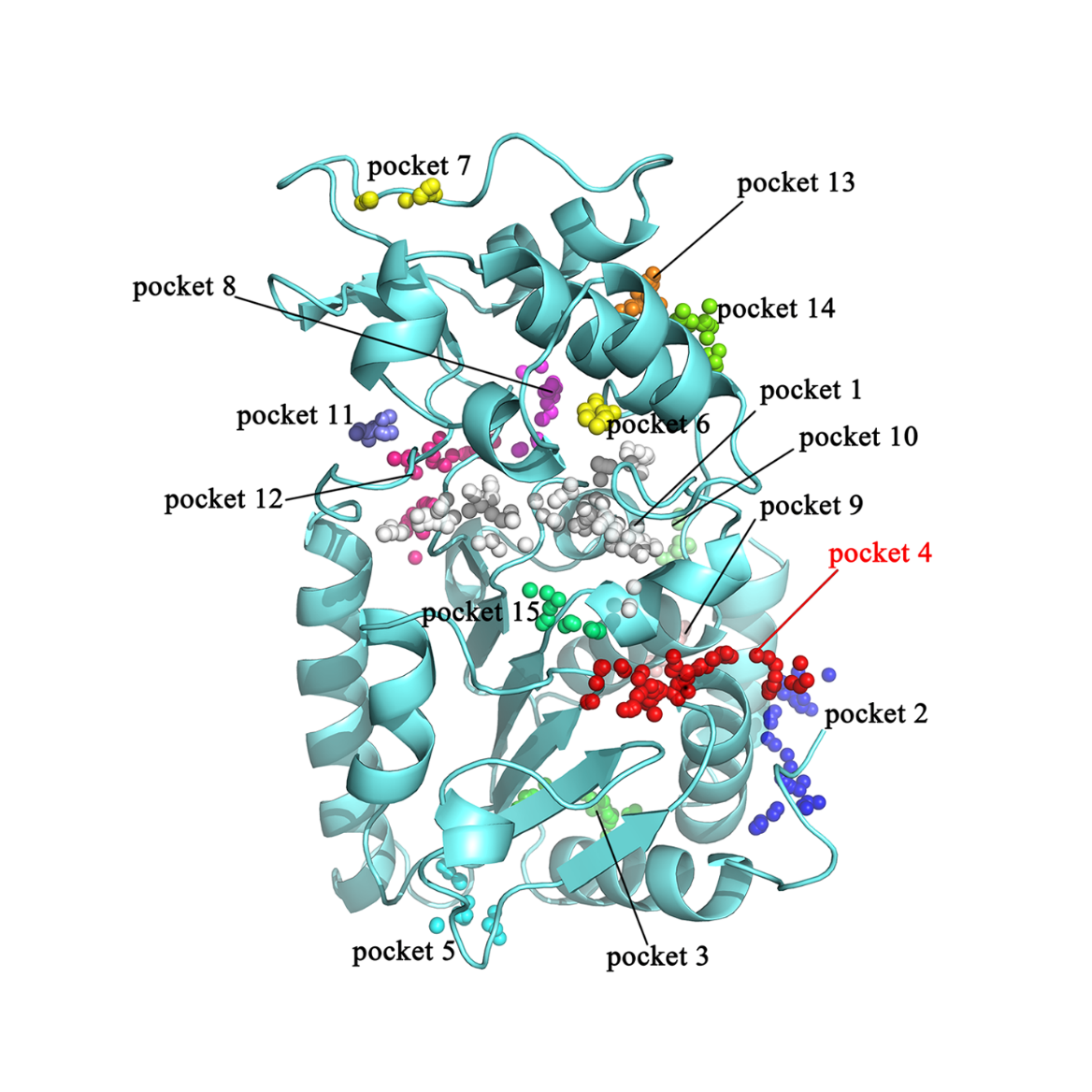
**

**Fig I in S1 Text: Overall distribution of 15 potential binding pockets predicted by Fpocket, with pocket 4 highlighted in red.**

**Table A in S1 Text: Top ten small molecules ranked by molecular docking affinity and their statistical parameters.**

| Ligand | Affinity(kcal/mol) | Mean | SD |
| --- | --- | --- | --- |
| 1569826 | -6.2 | -5.75 | 0.45 |
| 27546626 | -6.2 | -5.75 | 0.45 |
| 33961610 | -6.5 | -5.65 | 0.85 |
| 2151 | -6.1 | -5.45 | 0.65 |
| 967329 | -5.9 | -5.35 | 0.55 |
| 34067196 | -5.7 | -5.35 | 0.35 |
| 98044072 | -5.9 | -5.3 | 0.6 |
| 1718979 | -6 | -5.25 | 0.75 |
| 30678613 | -5.9 | -5.25 | 0.65 |
| 5385286 | -5.7 | -5.25 | 0.45 |


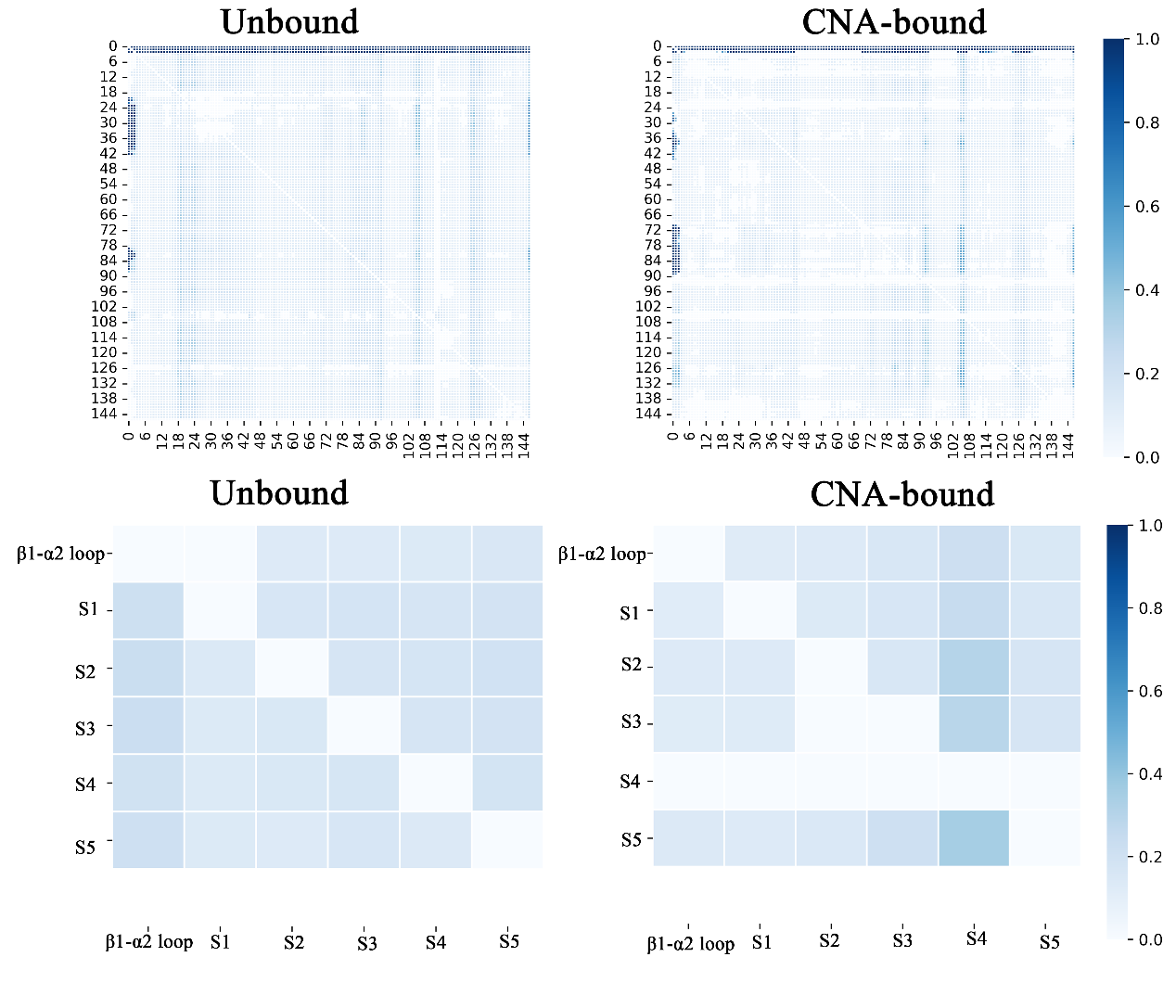


**Fig J in S1 Text: Control NRI Model Analysis.** To validate the reliability of constructing the NRI model using half of the residues, a control NRI (NRI⁻) model was built using the previously unselected half of the residues. (a, b) Residue–residue interaction matrices for the apo and CNA-bound states. (c, d) Inter-domain interaction strengths between the catalytic β1–α2 loop and the distal module. The results show that the trends observed in the control NRI model are consistent with those from the original model, further supporting the reliability of the original NRI model.
