# Supplementary material for: Carba-NAD binding activates SIR2 by reshaping conformational plasticity and rewiring long-range allosteric networks
Source: PLoS Comput Biol. 2026 Feb 20;22(2):e1013966. doi: 10.1371/journal.pcbi.1013966 (PMC12923025; doi:10.1371/journal.pcbi.1013966)
Supplement: Attachment — Submitted filename: Response to Reviewers.docx [file pcbi.1013966.s003.docx]

**Dear Editor:**

We are pleased to submit our revised manuscript entitled *“*Carba-NAD Binding Activates SIR2 by Reshaping Conformational Plasticity and Rewiring Distal Allosteric Networks*”* to *PLOS Computational Biology* for your consideration.

We sincerely thank you and the reviewers for the thoughtful, constructive assessment of our manuscript. We acknowledge that the initial submission required stronger methodological transparency, more robust sampling, and clearer, more concise presentation. Guided by the reviews, we have carried out substantial scientific and editorial revisions, which we summarize below.

1. **Robustness through independent replicates and physiological control** : For both apo and cofactor-bound SIR2 we added two independently seeded 3-μs runs, yielding three replicates (9 μs) per condition; key observables and mechanistic signatures reproduce across replicates. We further constructed and simulated the physiological NAD⁺ complex (three 3-μs replicates) under the identical protocol, which recapitulates the “loop-locking and periphery-releasing” mechanism seen with Carba-NAD.
2. **Methodological refinement**: To avoid trajectory-specific artifacts, each replicate is treated as an independent experiment in NRI, and inferred graphs are averaged across replicates. We expanded Methods to specify missing residues in homology completion; MD parameters (solvation, ions, cutoffs, restraints, thermostats/barostats, output cadence); NRI subsetting rationale and downsampling windows/stride; optimizer and training schedule; and docking setup. These details facilitate independent reproduction.
3. **Refined mechanism across scales (conformation → network → paths)**: With the expanded datasets, we sharpen how cofactor binding reweights the interaction topology—reducing the β1–α2 loop’s centrality, elevating distal relay hubs—and how shortest paths establish hierarchical routes (e.g., Pro214, Thr224) that physically couple local loop anchoring to distal flexibility.
4. **Allosteric pocket and virtual screening**: We integrated fpocket with network hotspots to identify Pocket 4, overlapping a relay residue and a high-centrality hub. A curated ZINC20 fragment-like library (8,852 compounds) was filtered to 147 diverse candidates for AutoDock Vina screening. Several ligands scored –5.7 to –6.5 kcal/mol; the top compound (ZINC1569826) forms four H-bonds (CYS-247/ASN-248/SER-270/ASP-271). Pocket volume (~220 Å³) and polarity support ligand accommodation, offering a concrete entry point for allosteric activation strategies.
5. **Data availability and figure/table improvements**: Trajectories and analysis scripts are deposited in a public repository (details in Data Availability). We regenerated or enlarged figures where clarity was needed (e.g., clearer bound vs. unbound in Fig. 1; explicit units on color bars; larger interaction matrices and clarified directed-graph legends in Fig. 5) and added standard errors to Table 1 to prevent over-interpretation of small differences; the Discussion now treats them as trends corroborated by RMSF/FEL/NRI.
6. **Deep editorial rewrite for clarity and concision**:In response to the writing concerns, we streamlined paragraphs, removed repetition, tightened transitions, standardized abbreviations (defining “CNA” in the Introduction and legends), and harmonized references and typography. Figure legends were made self-contained so that results can be understood without lengthy textual repetition. These changes significantly improve readability while preserving rigor.

We believe the revised manuscript now provides a robust, ensemble-validated account of how NAD⁺-family cofactors remodel SIR2 dynamics and network architecture, together with a practical, reproducible workflow—from replicate MD to ensemble NRI to structure-guided screening—for discovering allosteric intervention sites. We respectfully resubmit the manuscript and the detailed point-by-point response, and we welcome any further suggestions.

Sincerely,
Peng Sang
Dali University
Dali, Yunnan, China

A point-by-point response to all reviewers’ comments

Reviewer (s)' Comments to Author:

Reviewer #1 The authors hypothesize that the cofactor binding Carba-NAD, a non-hydrolyzable analog of NAD⁺) leads to long-range allosteric activation of SIR2. They proposed that this activation does not require “large-scale structural changes”, but rather involves redistribution of flexibility, local rigidification, and rewiring of interaction networks within the protein.
To do that they used 1 replica Long-timescale all-atom molecular dynamics (MD) simulations (3 μs for both apo and Carba-NAD bound states), using Neural relational inference (NRI), Principal component analysis (PCA) and free energy landscape (FEL) mapping for collective motions.
Carba-NAD binding “activates” SIR2 by rigidifying the catalytic β1–α2 loop, enhancing flexibility in distal regions, and rewiring residue interaction networks to enable long-range allosteric signaling through newly formed communication pathways.
There are some concerns about the methodology applied.
First, only 1 replica has applied in two systems. Good practices in MD simulations says that at least 3 replicas must be used to perform a standardized analysis of the dynamic of the system. (<https://pubs.acs.org/doi/10.1021/acs.jctc.8b00391>)

Response:

We are very grateful to the reviewer for raising this important concern regarding the limited number of MD trajectories in our original submission. We fully acknowledge that relying on a single trajectory per system may compromise statistical robustness, and we sincerely appreciate this constructive suggestion.

To address this issue, in the revised manuscript we have substantially expanded our simulations by performing two additional 3-μs unbiased MD trajectories for each system. This brings the total to three independent replicates per system, corresponding to 9 μs of aggregate sampling for each condition. All analyses presented in the revision are now based on these replicate trajectories. Importantly, the extended sampling yielded highly consistent outcomes with our original findings. Specifically, the RMSF profiles, free energy landscapes, and NRI-derived interaction networks reproduced the same mechanistic trends observed in the initial submission, confirming that our conclusions are not dependent on stochastic variations from a single run. By demonstrating reproducibility across independent replicates, the revised analysis provides a more rigorous statistical foundation and further reinforces the reliability of our mechanistic interpretations.

We have now explicitly clarified the use of three replicates in the Methods section, and have updated the Results descriptions and figure legends to indicate that the data shown are representative of consistent patterns across all replicates. We believe these additions substantially strengthen the robustness of our study, and we are sincerely grateful to the reviewer for encouraging us to improve this aspect.

Second, the natural substrate NAD+ has not been simulated neither analyzed, is there any difference between NAD+ and CNA? Does the natural substrate provide different behavior?

Response:

We sincerely thank you for raising the important concern regarding our use of Carba-NAD (CNA) instead of the natural cofactor NAD⁺. We fully recognize that this point is crucial for the mechanistic interpretation of our study. As you rightly noted, NAD⁺ is the physiological substrate, and clarification of our choice was needed.

In our original submission, CNA was employed because all currently available high-resolution crystal structures of yeast SIR2 in complex with a cofactor are solved with CNA rather than NAD⁺ (e.g., PDB ID: 1SZC). The native NAD⁺ is readily consumed during catalysis and therefore difficult to capture in stable complexes, whereas CNA, a non-hydrolyzable analog, preserves the canonical binding geometry of NAD⁺ while providing enhanced stability. To avoid any confusion to readers, we have revised the Introduction of the manuscript to explicitly explain why CNA is widely used in structural studies of sirtuins and why we employed it in our simulations.

At the same time, we fully agree with your suggestion that, although experimentally challenging, NAD⁺-bound complexes can indeed be computationally constructed and simulated. This is an excellent point that significantly strengthens the rigor of our study, and we are grateful for this advice. Accordingly, in the revised manuscript we have built an NAD⁺-bound complex via molecular docking and performed three independent 3-μs MD simulations (9 μs in total). The results from these NAD⁺ simulations showed highly consistent outcomes with those obtained for CNA.

Few minor errors must be address:
Reference literature must be standardized, not all the references are with the same style.
Figure 3 cbar has not dimensions.
Trajectories must be provided (<https://www.nature.com/articles/s42003-023-04653-0>)

Response:

We sincerely thank the reviewer for pointing out these important corrections, which have helped us to improve the rigor and clarity of the manuscript.

1. **Reference formatting:** We carefully re-examined the entire reference list and standardized the format according to the journal guidelines. All entries now follow a consistent style in terms of author names, journal abbreviations, volume, page numbers, and DOI inclusion.

**2.Color bar in Figure 3:** We have revised Figure 3 to include a properly annotated color bar with explicit dimensional units, thereby clarifying the physical meaning of the scale and improving figure interpretability.

**3.Trajectory files:** We fully agree with the reviewer that making raw simulation data available is important for transparency and reproducibility. Following this principle and in line with recent best practices (e.g., Nat. Commun. Biol. 2023, 6:4653), we have deposited all MD trajectories in a publicly accessible repository. The Data Availability section of the revised manuscript now includes the link and detailed instructions for access. These files cover all replicates of the apo and CNA/NAD⁺-bound systems, thereby ensuring that our analyses can be independently validated.

Reviewer #2: The manuscript by Zhang et al. describes the computational analysis of SIR2, an NAD+-dependent deacetylase involved in cellular aging. They conducted 3μs Molecular Dynamics (MD) simulations in combination with other techniques and subsequent analyses with the aim of exploring the changes in protein conformation & stability upon co-factor binding. Overall, the research is novel, the experiments conducted appear sound and appropriate, and the figures are clear, informative and well-illustrated with some minor comments.
The authors identify that upon CAN binding, the protein undergoes regional changes in rigidity and motility, with the binding region becoming more rigid and some key distal nodes increasing in flexibility. These observations are indeed novel, but, the manuscript presents some major caveats.

(1) Firstly, it would benefit from some greater robustness in the reporting of experimental procedures, as some key methodological details are missing. Secondly, and most importantly, the manuscript would benefit from a more concise writing style. Indeed, the arguments come across as drawn out and overcomplicated, and appear to be repeated throughout the manuscript. Unfortunately, the extent of this phenomenon drastically affects the clarity and ease of reading of the manuscript, thus reducing the impact & accessibility of the research.

Response:

We sincerely thank the reviewer for this insightful observation. We recognize that the original submission lacked certain methodological details and that our writing style was occasionally repetitive, which could compromise both reproducibility and clarity. We are grateful for your constructive feedback, which allowed us to improve the manuscript on both fronts.

In the revised version, the Methods section has been substantially expanded to include all essential simulation parameters—such as force field specification, solvation and ionization protocols, equilibration steps, thermostat and barostat settings, cutoffs, and trajectory output frequency—thereby ensuring transparency and reproducibility for future studies.

At the same time, we carefully revisited the writing style throughout the manuscript. While our initial intent was to maintain a tightly connected narrative, we recognize that this occasionally resulted in redundancy and overly extended transitions. We have therefore streamlined the text: overlapping arguments were consolidated, explanatory passages shortened, and transitions restructured for smoother flow. In addition, figure legends and tables were expanded so that key findings can be grasped more directly without lengthy textual repetition.

Together, these revisions have improved the clarity, accessibility, and scientific rigor of the manuscript, and we are grateful to the reviewer for guiding us in strengthening both the methodological robustness and the readability of our work.

(2) This also highlights that there may not be enough data presented to warrant a full article, and whether I) the data should be published under a different format, or II) further experiments (in vitro or in silico) are required before being published as a research article.

Response:

We sincerely thank the reviewer for raising this critical point. We fully recognize that in its original form, our study might have appeared limited in scope, and we greatly appreciate the reviewer’s emphasis on the need for both robustness and breadth to justify publication as a full research article. In revising the manuscript, we have therefore made substantial additions at multiple levels, aimed at consolidating the scientific rigor, reproducibility, and translational relevance of our work.

a) To strengthen statistical robustness, we extended our simulations by performing two additional replicates for each system. As a result, both the apo and CNA-bound ensembles are now supported by three independent 3-μs trajectories each (9 μs per system). All analyses in the revised manuscript are based on these replicate trajectories, and the results consistently reproduce the mechanistic signatures reported in the original submission. This expansion ensures that our conclusions are not dependent on a single trajectory, but are statistically reproducible across independent runs.
b) To address the potential concern that our conclusions could be biased by the use of the non-hydrolyzable analog Carba-NAD (CNA), we constructed and simulated the physiological NAD⁺-bound complex under an identical protocol. Again, three independent 3-μs trajectories (9 μs total) were performed. The NAD⁺ simulations recapitulate the key mechanistic features observed with CNA: rigidification of the β1–α2 loop, selective flexibilization of distal modules (S1–S5), decentralization of the catalytic loop as a signaling hub, and the emergence of relay residues such as Pro214 and Thr224 that establish multi-hop communication paths. These results are presented in the Supplementary Information and discussed in the main text, and they demonstrate that the allosteric mechanism uncovered here is intrinsic to the SIR2 scaffold and not a peculiarity of CNA.
c) To increase the translational relevance of our work—as suggested by the reviewer—we extended our study to include allosteric pocket prediction and virtual screening. Using fpocket, we identified potential cavities and cross-referenced them with graph-theoretical centrality analyses, thereby selecting a pocket overlapping with key network hubs. We then performed a fragment-based virtual screen by curating 8,852 fragment-like compounds from the ZINC20 database, applying stringent filtering and diversity selection to obtain 150 representative candidates, and docking them into the identified pocket using AutoDock Vina. The top-scoring compounds formed specific and consistent interactions within the cavity, providing a first set of chemical hypotheses for rational allosteric modulation of SIR2.
d) To complement the qualitative network rewiring analyses in the main text, we added graph-theoretical quantifications (e.g., degree and betweenness centrality distributions) in the revised manuscript. These additional metrics provide a more rigorous quantitative foundation for the reported reorganization of residue-residue communication.

Together, these expansions yield a study that now comprises 27 μs of aggregate sampling across three systems (apo, CNA, NAD⁺), replicate-based validation of all key observables, a physiological control confirming the generality of the mechanism, and an extension toward druggability assessment via virtual screening. We believe these additions elevate the work to the level expected of a full research article. We are deeply grateful to the reviewer for prompting these improvements, which have not only enhanced the rigor and reliability of our conclusions but also broadened the significance of the study from a mechanistic investigation to one with tangible translational implications.

(3) In particular, the authors state the data presented forms the basis for rational drug design; the inclusion of docking experiments with potential small compounds or proposed peptides for example would greatly increase the impact and pertinence of this research.

Response:

We sincerely thank the reviewer for this insightful suggestion, which has greatly helped us to strengthen the translational value of our work. We fully agree that establishing a direct link between the structural mechanisms uncovered in our simulations and the potential for rational drug design is essential to enhance the pertinence and impact of this study.

In response, we extended our analysis beyond conformational dynamics and network rewiring to evaluate whether the hotspots identified by NRI could also coincide with structurally accessible cavities. Using fpocket, we identified 15 candidate pockets on the SIR2 surface. When cross-referenced with residues exhibiting high betweenness centrality and path-based relay functions, Pocket 4 emerged as particularly notable. This cavity not only displayed favorable physicochemical properties for ligand accommodation, but also happened to encompass residues highlighted in our network analysis, thereby linking geometric accessibility with functional importance.

To probe the druggability of this cavity, we curated a fragment-like library of 8,852 chemically diverse compounds from the ZINC20 database. Following systematic filtering, 147 candidate molecules were subjected to molecular docking using AutoDock Vina. The results revealed that Pocket 4 can robustly accommodate diverse ligands, with binding energies in the range of –5.7 to –6.5 kcal/mol. Notably, the top-scoring compound (ZINC1569826) achieved a docking score of –6.2 kcal/mol and established four hydrogen bonds with residues CYS-247, ASN-248, SER-270, and ASP-271. These interactions not only confirm the chemical compatibility of the cavity but also underscore its potential as a bona fide allosteric site.

From a functional standpoint, two aspects of potential relevance can be highlighted. First, they provide independent evidence that the allosteric residues highlighted by our network analysis are indeed embedded within druggable cavities, thereby bridging structural dynamics with pharmacological feasibility. Second, unlike the orthosteric NAD⁺ site—which is highly conserved and typically yields inhibitors rather than activators—Pocket 4 offers a viable route to design allosteric activators of SIR2. Such molecules could mimic or enhance NAD⁺-mediated effects, potentially restoring deacetylase activity under conditions of NAD⁺ decline, and thus hold promise for the rational development of anti-aging therapeutics.

These results and their implications have been incorporated into the Results and further contextualized in the Discussion, thereby strengthening the translational perspective of our work. We are grateful to the reviewer for prompting this important extension of our work.

Comments:
Lines 16 & 17: There is no need to include these symbols as ‘¶’ only appears once when it indicates authors than contributed equally, while ‘&’ is not used in the author list.

Response:

We have removed the unnecessary symbols "¶" and "&" from the manuscript. Now, the author information section only retains essential notations, and the format has been fully standardized.

Line 54 & Figure 1 legend: The abbreviation CNA was only defined in the abstract, so should be defined in the introduction. The abstract should be considered independent to the main body of the manuscript with respect to abbreviation defining.

Response:

Following your suggestion, we have defined "CNA" upon its first appearance in the Introduction, while retaining its definition as an acronym in the Abstract. This ensures that readers can clearly understand the abbreviation's meaning even if they read the main text independently.

Figure 1: It appears that the unbound and bound images were generated from the same structure, as the only difference between the two panels is the addition of the ligand. If a change is present, it is not visible to the extent suggested in the manuscript to this point, and the figure should be adjusted to better display it.

Response:

We thank the reviewer for this helpful suggestion. In the revised manuscript, we have regenerated the structural diagrams for both the unbound and bound states. The updated figures now highlight the conformational differences between the two states more clearly, providing a more intuitive representation of how ligand binding influences the protein architecture.

Line 67: Missing space before “Despite”

Response:

We have added the missing spaces and corrected the typesetting issues.

Line 107: Which residues are missing? These should explicitly mentioned as the extent and position of these gaps is relevant to the quality of the resulting homology model.

Response:

We have clearly specified the missing residues to facilitate better understanding and reproduction by readers.

Line 186: Similarly, this residue subset should be detailed as this is a choice which will greatly impact the subsequent analyses. The rationale behind this selection should be explained.

Response:

Thank you for the reviewer's comments. Due to the excessive computational demands of the NRI model when dealing with full-length proteins of approximately 300 residues, training could not be completed within a reasonable timeframe. Therefore, we selected a representative subset of residues. The specific method involved selecting one residue at regular intervals, thereby retaining approximately half of the total residue count. This strategy effectively reduces computational complexity while generally preserving the representativeness of the protein structure. We have now added the justification for this selection and the specific methodology in the main text.

Line 189: What was the downsampling strategy? The chosen parameters could greatly affect the resulting sample sets and analyses.

Response :

Thank you for the reviewer's suggestion. The downsampling strategy we employed in our analysis references the work of Zhu et al., and we have provided a clear citation in the main text. Additionally, we have detailed the parameter settings for downsampling in the methods section to ensure the transparency and reproducibility of our analysis.

Table 1: The nature of the number in brackets should be specified. Presumably they are a form of error measurement, but the exact type should be specified.

Response :

We have clarified the meaning of the values in parentheses, indicating that they represent the standard error. This provides a clearer reflection of the variability and robustness of the results.

Figure 5: Panels B & C could benefit from being larger for better inspection of the interaction matrices. More detailed legend for panels D & E should be included, specifically explicitly mentioning the difference between the twinned graphs (i.e. to and from the β1-α2 loop)

Response :

We have revised Figure 5 as follows: Panels B and C have been enlarged to show the details of the interaction matrices more clearly. More detailed explanations have been added to the legends of Panels D and E, specifically clarifying the differences in the β1–α2 loop between the two paired figures, to help readers more easily understand the comparative results.

Reviewer #3: In this manuscript, the authors propose a novel mechanism by which the binding of a co-factors (Carba-NAD, an analogue for NAD+) induces conformational changes in Sirtuins at 2 levels. It decreases the flexibility around the catalytic site, and at the same time it increases flexibility at distal sites. The authors go further to describe in more detail how the allosteric signal propagates through the structure of the protein. The authors used MD simulations of the apo and cofactor-bound yeast SIR2 to train a Neural Relational Inference (NRI) which is a graph-based deep learning model to infere long range dynamic interactions between protein residues.
My main concern is that the entire manuscript is built based on a single MD simulation, 3 microseconds long for the unbound SIR2 and one more for the co-factor bound SIR2. The authors also do not provide any evidence that the 3 microseconds are enough to converge the motions and this is unlikely to be the case. Therefore it is not clear if the results presented have any statistical significance.

Response :

We sincerely thank the reviewer for raising this important point regarding the adequacy of sampling. To address this concern, we expanded our dataset beyond the original trajectories by performing two additional independent 3-μs simulations for both the apo and CNA-bound systems, resulting in a total of 9 μs per condition. Each trajectory was initiated with distinct velocity seeds to ensure statistical independence.

Across these independent replicates, we observed highly consistent structural trends, including rigidification of the catalytic loop, enhanced flexibility at distal regions, and the emergence of distributed relay nodes. These reproducible patterns strongly suggest that our observations are not artifacts of a single trajectory but reflect robust features of the underlying dynamics. Furthermore, multiple analyses—including RMSD stabilization, root-mean-square fluctuations, and free-energy landscape (FEL) projections—showed comparable distributions across replicates, indicating that the systems reached a stable equilibrium regime within the simulated timescale.

To further reinforce the generality of our conclusions, we extended the same protocol to the NAD⁺–bound system, which yielded results in excellent agreement with those obtained for CNA. The fact that two distinct cofactors, examined across independent replicates, consistently produced the same “loop-locking and periphery-releasing” mechanism provides strong support for the robustness of our findings. While we recognize that longer trajectories and enhanced-sampling strategies could capture additional rare events, the reproducibility across multiple independent runs and systems lends confidence that the mechanisms described here are reliable within the simulated timescale. We have revised the manuscript to clarify these methodological details and explicitly highlight the inclusion of replicate simulations.

The training of the NRI model is also done based on the same single simulations without any proof or test that the model captures converged long-range interactions in the dynamic network framework.

Response :

We sincerely thank the reviewer for raising this important point regarding the reliability of NRI training. We fully agree that relying on a single trajectory could compromise the statistical robustness of the inferred networks. To address this concern, we expanded our analysis beyond a single dataset. Specifically, for both the apo and CNA-bound systems, we generated three independent 3-μs simulations initiated from distinct velocity seeds. In the NRI framework, each trajectory was treated as an independent “experiment,” and the final edge weights were obtained by averaging across replicates. This setup ensures that the learned interaction patterns reflect reproducible dynamic couplings rather than idiosyncratic fluctuations of any individual trajectory.

The results from the three replicates were highly consistent, with all runs converging on the same set of relay residues (e.g., Pro214, Thr224) and high-centrality hubs (residues 252–254) that form the backbone of long-range signal propagation. Importantly, extending the same protocol to the NAD⁺–bound system reproduced the “loop-locking and periphery-releasing” mechanism identified with CNA, further underscoring the robustness of our findings across distinct cofactors.

Taken together, this ensemble-based training strategy significantly improves the reliability of the NRI model and provides strong confidence that the dynamic couplings we report are not artifacts of limited sampling but rather reproducible features of SIR2’s allosteric architecture. Corresponding clarifications have been added to the Methods section to explicitly describe this averaging procedure and its rationale.

In addition, there are very small variations between the 2 simulations reported in Table 1 and there are overinterpreted.

Response :

We sincerely thank the reviewer for pointing out the risk of overinterpreting the relatively small numerical differences reported in Table 1. We have carefully re-examined these results and now present them with greater caution. Specifically, we emphasize in the revised text that these changes should be regarded as indicative trends rather than definitive mechanistic evidence. To better convey the statistical robustness, we have added standard errors to Table 1 and explicitly reported them in the main text. Importantly, we also adjusted the discussion to clarify that, while the absolute magnitudes of the numerical shifts are limited, they are consistent with the broader conformational and network-level changes observed across independent analyses (RMSF, FEL, NRI). Thus, our conclusions are not based on isolated numerical differences, but on convergent evidence from multiple complementary approaches.
