# Supplementary material for: Carba-NAD binding activates SIR2 by reshaping conformational plasticity and rewiring long-range allosteric networks
Source: PLoS Comput Biol. 2026 Feb 20;22(2):e1013966. doi: 10.1371/journal.pcbi.1013966 (PMC12923025; doi:10.1371/journal.pcbi.1013966)
Supplement: Attachment — Submitted filename: Response_to_Reviewers_auresp_2.docx [file pcbi.1013966.s004.docx]

**Dear Editor,**

We are pleased to submit our revised manuscript entitled *“*Carba-NAD Binding Activates SIR2 by Reshaping Conformational Plasticity and Rewiring Distal Allosteric Networks*”* to *PLOS Computational Biology* for your consideration.

We sincerely appreciate the opportunity to revise our manuscript. We are especially grateful to the reviewers for their time and effort in the second-round evaluation, particularly for their valuable suggestions regarding the rigor of data analysis, figure readability, and reproducibility of results. We recognize that the previous version of the manuscript had shortcomings in methodological description and data statistics. Accordingly, we have carefully considered each comment and performed extensive additional analyses and thorough revisions throughout the manuscript. We believe that, after incorporating these constructive suggestions, the logical flow is stronger and the evidence supporting our conclusions is more robust.

In response to the reviewers’ feedback, we have undertaken substantial scientific and editorial improvements, summarized as follows:

**1.Enhanced methodological transparency and clarified analysis strategies:**
We have added details regarding the force field parameters for the structural Zn²⁺ to ensure simulation accuracy. Additionally, we clarified our treatment of replicate simulation data: while RMSD and Neural Relational Inference (NRI) model analyses were performed on independent trajectories for validation or ensemble averaging, all other analyses—including RMSF, free energy landscape, PCA, etc.—were performed on concatenated trajectories to fully exploit the sampling data.

**2.Improved readability through comprehensive rewriting:**
Based on suggestions regarding manuscript readability, we extensively revised and polished the text. Paragraph structures were reorganized, redundant content removed, logical transitions strengthened, and terminology and abbreviations standardized. These changes maintain scientific rigor while significantly enhancing the clarity and flow of the manuscript, allowing readers to more easily understand the complex biophysical mechanisms.

We believe that the revised manuscript not only provides a robust mechanistic explanation of how NAD⁺-family cofactors reshape SIR2 dynamics but also presents a standardized workflow from repeated MD simulations to ensemble NRI analysis and structure-guided screening.

We respectfully resubmit the manuscript along with detailed point-by-point responses and look forward to your further guidance.

Sincerely,
Peng Sang
Dali University
Dali, Yunnan, China

A point-by-point response to all reviewers’ comments

Reviewer's Responses to Questions

Reviewer #1: The authors have satisfactorily addressed all inquiries raised in the previous round of peer review and have incorporated the suggestions provided by the referees. The authors have addressed all inquiries raised during the first round of peer review and have incorporated all referees’ suggestions in a thorough and constructive manner. It now follows goof MD practices applying 3 replicas. The proposed revisions enhance clarity and strengthen the evidentiary basis of the conclusions, fitting with the PLOS Computational Biology standards. I would like to congratulate again the authors for this complete research.

Response: We sincerely thank you for the recognition and encouragement you have given our work in this round of review. We are deeply grateful for the valuable suggestions you provided in the previous rounds. Your comments have not only helped us improve our analytical approaches but have also greatly enhanced the logic, clarity, and scientific rigor of the manuscript. During the revision process, we strictly followed the guidelines of PLOS Computational Biology, substantially strengthening the number of simulation replicates, analytical procedures, and presentation of results, and we carefully incorporated all constructive comments from you and the other reviewers. We truly appreciate your acknowledgment of our efforts in employing three independent simulations, improving figure readability, and reinforcing the evidential support for our conclusions. Your recognition is a tremendous encouragement to us. Once again, we sincerely thank you for your professional dedication and patient guidance throughout the entire review process.

Reviewer #3: I appreciate very much the effort the authors put into addressing my original comments. Indeed, the revised manuscript contains now a significantly increased amount of data which the authors claim to support their main conclusions. The authors provide now 3 independent MD simulations for each condition and they also include simulation with the naturally occurring co-factor NAD+.

However, it remains unclear how the authors used these new data. With the exception of Figure 2 in which they plot the RMSD individually for each trajectory, it remains unclear how the authors calculated all other parameters using all data they generated. E.g. they do not specify whether they used the ensemble of simulations to calculate the numbers reported or they present averages from the 3 independent replicas. If the latter, error bars with ranges are missing in plots and table. Without a clear explanation of how the authors incorporated all simulations in their analysis, it is impossible to assess the reproducibility and variability between different simulations.

Even if the authors put some effort into improving readability, the paper is still quite bulky and hard to read but in the same time its missing important methodological details.

Response:We are very grateful for your positive evaluation of our previous revision, and we sincerely appreciate your highly perceptive identification of the gaps in our data processing and statistical descriptions.We fully agree with your point: simply increasing the amount of data is not sufficient—clearly explaining how the three independent simulations were integrated is essential for evaluating the reproducibility of the work. We apologize for not articulating this clearly in the previous version.In response to your question, we would like to clarify our analysis strategy:1.Regarding the RMSD analysis:We indeed analyzed the three trajectories independently. The purpose was to verify the stability and convergence of each individual simulation (as shown in Fig. 2).2.Regarding all other analyses (RMSF, PCA, FEL, contact analysis, etc.):We employed a trajectory concatenation approach. The three independent simulations were concatenated into a single continuous “meta-trajectory.”Rationale for this approach:Although we performed three independent repeats to avoid trapping in local minima, we acknowledge that a sample size of *n* = 3 is statistically limited. Calculating averages and error bars based solely on these three data points would not provide robust statistical meaning and could even be misleading.Therefore, instead of averaging across the three runs, we chose to merge them to maximize the sampling of the conformational space. This strategy allows analyses such as PCA and FEL to capture the cumulative dynamical behavior of the system, rather than emphasizing the variance across a small number of runs.

In addition, regarding your comment that *“the manuscript remains somewhat lengthy and not sufficiently easy to read,”* we have implemented systematic improvements. We reorganized and streamlined the overall structure and paragraph flow, removed repetitive descriptions, and enhanced the clarity of the figures and Methods section. These revisions were made to improve overall readability and help readers more easily follow the main arguments and analytical framework.

Some more specific comments:

From the Methods, it remains unclear whether the independent simulations were started after the equilibrations by just re-assigning the velocities or if the entire equilibrations were performed for each replica.

Response:We are very grateful to the reviewer for raising this crucial methodological question. We apologize for the earlier ambiguous description, which failed to clearly define the independence of our simulation replicates. We fully agree that the manner in which replicates are initialized is fundamental to sampling reproducibility and statistical validity.To address your concern and to ensure the highest standard of *uncorrelated sampling*, we wish to clarify that each of the three replicates underwent a completely independent equilibration procedure, rather than merely reassigning velocities to the same pre-equilibrated structure.We adopted this stringent protocol to ensure that the replicates diverge as early as possible in the simulation workflow. This strategy minimizes bias introduced by a single equilibration trajectory and maximizes exploration of conformational space.We have rewritten the Methods section to describe the protocol in detail. The revised text reads as follows:To obtain statistically independent sampling, each replicate was subjected, after the initial equilibration, to a fresh reassignment of initial velocities and then independently equilibrated with an additional 100 ps NVT followed by 100 ps NPT (using the same temperature, pressure, and positional restraint settings). Production simulations of 3 μs were then started from these independently equilibrated states.We hope this detailed explanation clarifies our simulation protocol.

It is not clear what Zn2+ parameters were used

Response:We thank the reviewer for raising questions regarding the source and treatment of the Zn²⁺ parameters. We provide further clarification as follows: the structural Zn²⁺ in SIR2 is located at a conserved Cys₄ coordination site, coordinated by four cysteine residues in a typical tetrahedral geometry. This Zn²⁺ serves solely to maintain local structural stability and does not participate in the catalytic process.During system preparation, the parameters and topology for Zn²⁺ were automatically generated by CHARMM-GUI using the metal ion modeling protocol within the CHARMM36 force field. This protocol can automatically recognize metal ions and their coordinating residues during system setup, and consistently integrate the corresponding parameters and topology into the final protein topology. Therefore, the Zn²⁺ parameterization and its coordination environment were fully determined and consistent at the construction stage.We have added this clarification in the Methods section of the revised manuscript to provide readers with a clearer understanding of how Zn²⁺ is handled in the force field.

Also in Methods: the authors mention they calculate residue-residue contacts but given the data it seems that what they report are actually atom-atom contacts. Also not clear if only heavy atoms have been used for the calculation.

Response: We sincerely thank the reviewer for carefully examining our Methods section and for raising this important point. Regarding your observation that our description of “residue–residue contacts” and the presented results are more consistent with “atom–atom contacts,” and the lack of clarity on whether only heavy atoms were used, we provide the following clarification:In this study, we actually calculated contact numbers based on all-atom interactions (including hydrogen atoms), rather than limiting the analysis to heavy atoms. Hydrogen atoms constitute approximately half of the total atoms in proteins and play a critical role in defining the molecular surface and volume. Therefore, including hydrogen atoms allows for a more sensitive and comprehensive assessment of interfacial packing density and steric complementarity, and enables the capture of short-range interactions (such as van der Waals contacts involving protons) that might be underestimated when considering only heavy atoms. Specifically, whenever the distance between any two atoms was below the threshold of 0.45 nm, it was counted as an atom–atom contact.We acknowledge that the original phrasing of “residue–residue contacts” was inaccurate and may have caused confusion. In the revised manuscript, we have clarified and standardized the terminology to accurately reflect the analysis. We greatly appreciate the reviewer for pointing this out, as it significantly improves the transparency and precision of our Methods description. The relevant changes have been incorporated in the revised manuscript. Thank you again for your valuable suggestion.

Was the “FEL” abbreviation defined prior to its use at page 8 in the manuscript?

Response:We sincerely thank the reviewer for carefully examining the details of our manuscript. Regarding your comment on whether the abbreviation “FEL” was defined before its use on page 8, we confirmed that in the original manuscript the full term was indeed not provided at first occurrence. We have corrected this in the revised manuscript by defining it as free energy landscape (FEL) at its initial appearance and ensured consistent usage throughout the text. We greatly appreciate your careful attention, which allowed us to further improve the clarity of the manuscript.

In the sparse sampling approach for the NRI model, the authors use only half of residues,. This seems rather arbitrary. How do the author justify the selection of 50% of the residues ? They do not present any data to show that 50% would be sufficient to preserve dynamics and no data showing that even sparser choices (e.g. 25%) would not be enough for preserving dynamics anymore.

Response:We sincerely thank the reviewer for the careful attention and valuable comments regarding the sparse sampling strategy in the NRI model. Concerning your questions about whether using only 50% of residues is reasonable, sufficient to preserve system dynamics, and whether lower sampling fractions were evaluated, we provide the following clarification: First, the sparse sampling strategy employed in this study was not chosen arbitrarily; rather, it strictly follows the prior research findings of He et al. and Wang et al. (relevant literature has been cited in the revised manuscript). These studies explicitly demonstrate that when constructing graph-based molecular dynamics inference models, spatially uniform sampling of a subset of nodes is sufficient to adequately capture key system dynamics, with predictive accuracy remaining highly consistent with models utilizing all nodes. Based on this well-established methodological principle, we adopted a spatially uniform sampling strategy using 50% of the residues to construct the NRI model, thereby ensuring the reliability of the results. Second, to validate the robustness of sparse sampling for our specific system, we performed a control experiment. We trained an NRI model using the other 50% of residues that were not included in the initial sampling. The results from this control NRI model were highly consistent with those of the original NRI model, demonstrating that training on either half of the residues yields robust and reproducible dynamic structural patterns.These validation results are presented in the Supplementary Information (Figure S10), showing the agreement between the two sets of models in key structural modules, thereby providing direct evidence supporting the sparse sampling strategy. We greatly appreciate your important suggestion, which allowed us to further strengthen and validate the rationale for our model choice and provide the necessary supporting data.

In Fig 1 it is not clear if the three pictures are having the same orientation. If that is the case, it seems that the colors are not consistent between the 3 pictures. The beta1-alpha2 is shown in purple in the upper illustration and in pink below. Or maybe the labels are not placed in the correct places. In any case, quite unclear.

Response:We sincerely thank the reviewer for pointing out the potential confusion in Figure 1 regarding structural orientation and color coding.To address your concerns, we clarify the following: the three structures in Figure 1 are indeed presented in the same orientation. The main source of confusion arose because the β1–α2 loop was highlighted in purple in panel (a), whereas panels (b) and (c) originally used different color schemes, affecting overall consistency.Following your suggestion, we have revised Figure 1 as follows:1.The β1–α2 loop in panels (b) and (c) has been recolored to match the purple used in panel (a).2.The figure legend and caption now explicitly state that all three structures are displayed in the same orientation.3.We have also checked and ensured that all labels, positions, and colors are consistent to improve overall readability.We greatly appreciate your careful observation, which allowed us to further enhance the clarity and legibility of the figure.

Font text on graph labels are often too small and hard to read. For example, the RMSD plots in Figure 2 the Y range is too large therefore masking the differences between the replica simulations. Also, from the RMSD plots it is apparent that one of the simulations (in blue) with the co-factor bound behaves differently than the other two. However, the authors do not comment on that

Response:We sincerely thank the reviewer for the careful evaluation of the figure quality and RMSD analysis.In response to your comments, we have made the following revisions and clarifications:1.Text size in figures:We have readjusted the font size and layout of all figures to ensure good readability even before zooming in.2.Y-axis range of the RMSD plot in Figure 2:Following your suggestion, we reset a more appropriate Y-axis range to better display the differences among the three replicate simulations, allowing subtle variations between trajectories to be more clearly visible.3.Discussion of differences among replicate trajectories:You pointed out that, in the presence of the cofactor, one trajectory shows RMSD behavior distinct from the other two. We have conducted additional analysis and added a clear explanation in the figure legend:
*“In the CNA-bound state, the third trajectory (now shown in orange) exhibits higher RMSD fluctuations due to more pronounced local conformational rearrangements in the α1–α2 region, which were not observed in the other two trajectories. Despite the larger amplitude, these fluctuations remain within the stable conformational space and do not compromise the overall structural integrity.”*

We have updated the figure legend to include this explanation.We sincerely appreciate your valuable suggestions, which have significantly improved both the clarity of the figures and the completeness of our analysis.

It is not clear what it is shown in Table 1. I would assume that it is the average and standard deviation but this is not specified in the footnotes and values for the standard deviation appear too small. Also not clear how the values were calculated (e.g. considering all simulation replicas or is it an average based on the 3 simulations ?). Could the authors provide the time series of these data as supplementary information ?

Response:We sincerely thank the reviewer for the careful evaluation and valuable comments regarding the data presentation in Table 1.In response to your questions, we provide the following clarifications:1.Meaning of values in parentheses in Table 1:You noted that the numbers in parentheses seemed unusually small and questioned whether they represented standard deviations. Upon review, we confirm that the original manuscript reported standard errors; however, this was not clearly indicated in the table legend, leading to potential confusion. Following your suggestion, we have updated all values in parentheses to represent standard deviations, and we have clarified this explicitly in the main text.2.Calculation method of the values:We have now clearly stated in both the main text and the table legend that all metrics in Table 1 were calculated as averages and standard deviations over concatenated trajectories from three independent replicate simulations. Our previous description lacked clarity, and we appreciate your reminder to standardize it.3.Provision of time-series data:Following your recommendation, we have organized and added the corresponding time-series data for metrics such as NHB, contact number, SASA and Rg to the Supplementary Material (Figure S1), enabling reviewers and readers to verify trends and fluctuations.

We sincerely appreciate your suggestions, which have significantly improved the transparency, interpretability, and reproducibility of the results.

Figure 3: how was the RMSF calculated ? Considering all simulations as an ensemble or from the individual simulations ? If the later, the plot from which simulation is shown ?

Response:We thank the reviewer for the careful attention to the calculation of RMSF in Figure 3.To clarify: the RMSF values were calculated based on concatenated trajectories from the three independent simulations, rather than from a single trajectory. In other words, the backbone atom coordinates from the three replicate simulations were concatenated along the time dimension, and the RMSF was then computed over this combined trajectory, providing a more robust and representative fluctuation profile.We have explicitly clarified this point in the Methods section of the revised manuscript to avoid potential misunderstandings.We appreciate your valuable comments, which have helped us present the RMSF analysis more clearly.

And the last comment can be applied to principal component analysis and in fact all other analysis (see first comment above)

Response:We sincerely thank the reviewer for highlighting the importance of methodological consistency in our analyses. Regarding your questions about the calculation procedures for PCA and other analyses, we have provided comprehensive clarifications in the Methods section of the revised manuscript.Specifically, RMSD and NRI model analyses were performed separately for each of the three independent simulation replicates to preserve trajectory independence for comparative purposes. For all other structural analyses—including PCA, RMSF, SASA, Rg, and NHB—calculations were performed on concatenated trajectories of the three replicates, providing more comprehensive sampling and statistically robust results.In the revised manuscript, we have clearly indicated these calculation approaches in the relevant text and figure legends to avoid ambiguity and enhance methodological transparency. We greatly appreciate your insightful comment, which has allowed us to further improve the rigor and reproducibility of our study.
