# Supplementary material for: Carba-NAD binding activates SIR2 by reshaping conformational plasticity and rewiring long-range allosteric networks
Source: PLoS Comput Biol. 2026 Feb 20;22(2):e1013966. doi: 10.1371/journal.pcbi.1013966 (PMC12923025; doi:10.1371/journal.pcbi.1013966)
Supplement: Attachment — Submitted filename: Response_to_Reviewers_auresp_3.docx [file pcbi.1013966.s005.docx]

**Dear Editor,**

We sincerely thank you and the reviewers for your careful evaluation of our manuscript and for the valuable comments and suggestions. We greatly appreciate the time and effort invested by the reviewers, as their feedback has played an important role in improving the scientific quality and methodological rigor of our work.

In response to the reviewers’ comments, we have carried out a comprehensive and thorough revision of the manuscript. In particular, regarding the concern raised by Reviewer about the definition of contacts, we carefully re-examined our original analysis and fully adopted the reviewer’s suggestion by recomputing the relevant analyses. Specifically, we redefined and recalculated the native contacts by considering only heavy atoms (i.e., all non-hydrogen atoms) in the protein, while keeping the distance cutoff unchanged at 4.5 Å, and performed the contact analysis again. The corresponding results have been updated accordingly, and the methodological description has been clearly revised in the main text to eliminate ambiguity and improve clarity and consistency.

After the reanalysis, although the absolute values of the contact numbers changed, the relative trends among different systems remained consistent, further confirming the robustness of the main conclusions of this study. We believe that these revisions effectively address the reviewer’s concerns and significantly enhance the methodological rigor and reproducibility of the manuscript.

Once again, we sincerely thank the editor and the reviewers for their recognition of and guidance on our work. We hope that the revised manuscript now meets the publication requirements of the journal, and we look forward to your further instructions.

Sincerely,
Peng Sang
Dali University
Dali, Yunnan, China

response to reviewers’ comments

Reviewer #3: I appreciate that the authors have put significant effort in addressing all points I raised. The paper has improved to a point at which it could be published. One concern I still have: why do the authors use 4.5 A as threshold for contacts involving hydrogen atoms ? Since bonds involving hydrogen are rigid during the simulations, I do not see the reasoning for including hydrogens in such calculations. Moreover, residue-residue contacts can be overestimated with such an approach. I would advise redoing the contact analysis by just including heavy atoms and keeping the threshold of 4.5.

Response:

We sincerely thank the reviewer for this insightful and constructive comment. We appreciate the reviewer’s careful examination of the contact definition and for pointing out the potential implications of including hydrogen atoms in the close-contact analysis.

In the original analysis, NCICs were computed using the *gmx mindist* tool by selecting the “Protein” group for both interacting partners, which includes all protein atoms. As correctly noted by the reviewer, this definition allows hydrogen atoms to contribute to the minimum interatomic distance, and due to their small van der Waals radii and constrained motion under bond constraints (e.g., LINCS), this may lead to an overestimation of close interaction contacts.

We agree with the reviewer that, for the purpose of quantifying residue–residue proximity and avoiding artificial inflation of contact counts, a heavy-atom–based definition is more appropriate. Following the reviewer’s suggestion, we have therefore repeated the NCIC analysis by excluding all hydrogen atoms and considering only heavy atoms, while keeping the distance cutoff unchanged at 4.5 Å. In this revised analysis, a close interaction contact is defined when the minimum distance between any pair of heavy atoms from the selected protein groups is smaller than 0.45 nm.

Importantly, although the absolute number of NCICs is reduced when hydrogen atoms are excluded, the overall trends and comparative differences between the studied systems remain unchanged. This confirms that the main conclusions of the manuscript are robust with respect to the specific contact definition employed. The Methods section has been revised accordingly to clearly state that only heavy atoms were included in the contact analysis.

We thank the reviewer again for this valuable suggestion, which has helped us to improve the methodological rigor and clarity of the manuscript.
